# High‐Throughput Fabrication of Zero‐Mode Waveguide Nanoaperture Arrays with Sol‐Gel Nanoimprint Lithography for Enhanced Single Molecule Fluorescence Detection

**DOI:** 10.1002/smll.202510587

**Published:** 2025-11-21

**Authors:** Hamza Khelidj, Anthony Gourdin, Igor Ozerov, Antonin Moreau, Badre Kerzabi, David Grosso, Jérôme Wenger

**Affiliations:** ^1^ Aix Marseille Univ CNRS Centrale Med Institut Fresnel AMUTech Marseille 13013 France; ^2^ Solnil 163 Avenue de Luminy Marseille 13009 France; ^3^ Aix‐Marseille Univ CNRS CINaM AMUTech Campus de Luminy Marseille 13288 France

**Keywords:** nanofabrication, nanoimprint lithography, plasmonics, single molecule fluorescence, zero‐mode waveguide nanoaperture

## Abstract

Zero‐mode waveguides (ZMWs) are subwavelength metallic nanoapertures enabling enhanced single‐molecule fluorescence detection at micromolar concentrations in conditions far beyond the diffraction‐limited capabilities of confocal microscopes. However, their widespread use remains limited by the complexity and cost of the nanofabrication techniques, such as focused ion beam and electron‐beam lithography. Here, a scalable, cost‐effective, and high‐throughput method for fabricating high‐performance ZMW arrays is presented, which combining sol‐gel nanoimprint lithography (NIL) with hydrofluoric acid (HF) vapor‐phase etching. This approach enables the parallel fabrication and massive replication of ZMW nanoapertures with attoliter volumes, without requiring expensive equipment. The optical performance of the resulting ZMWs is validated through a series of single‐molecule fluorescence experiments, including burst analysis, fluorescence correlation spectroscopy (FCS), and single‐molecule Förster resonance energy transfer (smFRET). The ZMW nanoapertures demonstrate up to 8× fluorescence brightness enhancement, sub‐millisecond temporal resolution, and broadband spectral operation across the visible range. This method represents a significant advance in making nanophotonic devices more accessible, paving the way for a broader adoption of ZMWs in single‐molecule biosensing and integrated nanophotonic systems.

## Graphical abstract

The researchers developed a scalable and cost‐effective method to fabricate zero‐mode waveguide (ZMW) nanoapertures in metallic films using sol‐gel nanoimprint lithography and vapor‐phase etching. These nanostructures confine light to attoliter volumes, enhancing fluorescence brightness and enabling single‐molecule detection at micromolar concentrations. Their approach makes nanophotonic tools more widely accessible for biophysical research.

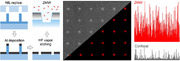

## 1 Introduction

Single‐molecule fluorescence techniques have become essential tools in modern biophysics to reveal the molecular mechanisms underlying biological processes.^[^ ^1^, ^2^, ^3^, ^4^, ^5^, ^6^, ^7^ ^]^ However, single‐molecule detection remains predominantly performed on confocal fluorescence microscopes constrained by diffraction, which in turn imposes two major fundamental limits.^[^ ^8^, ^9^, ^10^ ^]^ First, due to the size mismatch between the dimension of a single quantum emitter and the wavelength of light, the light‐matter interaction at the nanoscale is inefficient, leading to a weak detected fluorescence brightness of a few tens of thousands of photons per second.^[^ ^11^, ^12^ ^]^ This low signal not only challenges the detection of single‐molecule events but also limits the temporal resolution on diffraction‐limited microscopes,^[^ ^13^, ^14^, ^15^ ^]^ typically in the millisecond range in order to collect enough photons per time bin. The second major limit concerns the detection volume: with a typical confocal volume of 1 fL (10^−15^ L), diluted concentrations in the pico to nanomolar range are required to isolate a single molecule on a diffraction‐limited microscope. Such conditions are incompatible with most physiological and biochemical reactions, which occur at micromolar to millimolar concentrations.^[^ ^16^, ^17^, ^18^ ^]^

Both limitations of single‐molecule fluorescence detection can be simultaneously addressed by introducing zero‐mode waveguide (ZMW) nanoapertures,^[^ ^8^ ^]^ which are subwavelength holes milled into an opaque metallic film.^[^ ^18^, ^19^, ^20^, ^21^ ^]^ As light is confined into the ZMW nanoaperture,^[^ ^22^, ^23^ ^]^ the detection volume for single molecule sensing is drastically reduced below the diffraction limit,^[^ ^24^, ^25^, ^26^, ^27^, ^28^ ^]^ allowing to work at 1000× higher concentrations with an extended flexibility as the detection volume is no longer constrained by diffraction but instead defined by the ZMW design (diameter, thickness, material used). In contrast to total internal reflection fluorescence (TIRF) microscopy, which enables background reduction and single‐molecule detection for surface‐immobilized samples, ZMWs confine light in all three dimensions at the nanoscale, allowing single‐molecule measurements in freely diffusing conditions and at micromolar concentrations.^[^ ^26^, ^29^ ^]^ Moreover, the light‐matter interaction is enhanced inside the ZMW, leading to higher fluorescence brightness per molecule and improved temporal resolution.^[^ ^15^, ^30^, ^31^, ^32^, ^33^, ^34^ ^]^ ZMWs offer several additional practical advantages, notably an efficient rejection of the background from fluorescent molecules in the solution outside the aperture, a broadband spectral operation due to the absence of sharp resonances as compared to plasmonic nanoantennas,^[^ ^35^, ^36^, ^37^, ^38^ ^]^ and a straightforward implementation in confocal scanning microscopes.

Despite the large interest raised by the ZMW nanoapertures, their applications remain relatively limited in biophysics research^[^ ^29^, ^39^, ^40^, ^41^, ^42^, ^43^, ^44^, ^45^, ^46^ ^]^ and industrial settings.^[^ ^47^, ^48^ ^]^ This is primarily due to the complexity and high operational costs associated with their nanofabrication and the lack of commercial availability.^[^ ^49^, ^50^ ^]^ Direct milling into the metal film using focused ion beam (FIB) is a straightforward conceptual approach,^[^ ^51^, ^52^, ^53^ ^]^ but the serial nature of the FIB milling strongly limits the throughput: with a typical milling time of 0.5 s per ZMW, fabricating a 200 × 200 ZMW array already requires 5.5 h. Electron‐beam lithography (E‐beam) addresses this throughput limitation by enabling faster nanofabrication,^[^ ^54^, ^55^, ^56^, ^57^, ^58^ ^]^ yet the approach is restricted to shallow structures and often lacks the undercut into the glass substrate necessary to reach high brightness enhancement.^[^ ^32^, ^59^, ^60^ ^]^ Moreover, both FIB and E‐beam techniques require expensive instruments that are costly to purchase and to maintain. As a nanofabrication alternative, nanosphere lithography (NSL) with template stripping features a significantly reduced operation cost,^[^ ^61^, ^62^, ^63^, ^64^ ^]^ yet it has design limitations on the ZMW shape and spacing, requiring specific additional steps to produce regularly positioned arrays of ZMWs.^[^ ^50^ ^]^ Nanoimprint lithography (NIL) is a compelling alternative to replicate nanostructured patterns from a mold with low cost, high throughput, and high resolution.^[^ ^65^, ^66^ ^]^ Although NIL has now become a well‐established technique in electronics and photonics, its application to the fabrication of ZMW arrays remains largely unexplored.^[^ ^49^, ^67^ ^]^ This is mainly due to the challenges associated with producing high‐aspect‐ratio features and reliably opening subwavelength apertures in metal films.

Here, our aim is to overcome these limitations and bridge the technological gap between single‐molecule fluorescence and nanophotonics by developing a cost‐effective and scalable method to fabricate ZMW arrays of high optical performance. Our approach combines sol‐gel nanoimprint lithography (NIL)^[^ ^68^, ^69^ ^]^ for high‐throughput sample fabrication with hydrofluoric acid (HF) vapor etching^[^ ^70^ ^]^ to create the nanoapertures in a massively parallel fashion without requiring any expensive nanofabrication system (except for the initial master fabrication). This original approach enables nanometer‐scale resolution, large‐scale production, and low cost per sample. We validate the high optical performance of the resulting ZMW arrays through a series of single‐molecule fluorescence experiments, demonstrating attoliter detection volumes, enhanced brightness, and sub‐millisecond temporal resolution over a broad spectral range. While NIL and HF etching are widely used in microelectronics and microelectromechanical systems (MEMS), respectively, their combined application remains unprecedented in nanophotonics, opening a promising solution for large‐scale fabrication of high‐performance nanoaperture samples at moderate costs that can be used for various applications in plasmonics sensing and beyond.^[^ ^71^, ^72^, ^73^ ^]^

## 2 Results and Discussion

**Figure 1:**
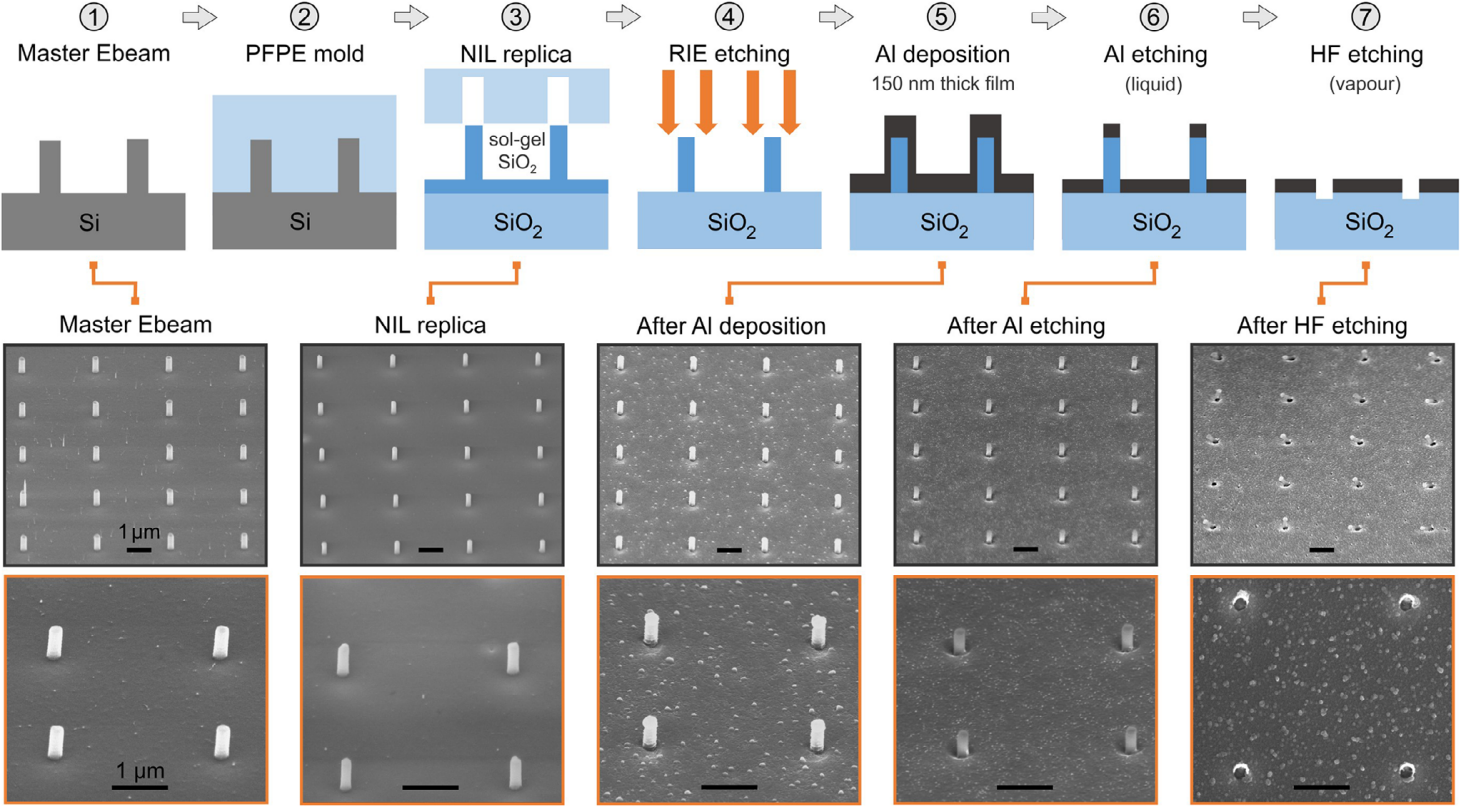
Zero‐mode waveguide nanoaperture fabrication methodology merging sol‐gel nano‐imprint lithography with HF vapor etching. See text for details of each step. The bottom scanning electron microscopy (SEM) images correspond to representative samples after the selected fabrication steps. The sample surface is tilted by an angle of 52° with respect to the SEM view direction; top‐view SEM images are provided in Figure S1 (Supporting Information). All the scale bars denote 1 µm length.

**Figure** **1** describes schematically our ZMW nanofabrication method combining sol‐gel NIL and selective acid etching of silicon dioxide versus aluminum in the vapor phase. The first step begins with the fabrication of an array of silicon nanopillars using E‐beam lithography, which will serve as a master structure to be replicated during the NIL process (see Experimental Section for details). For this work, the silicon nanopillars have a diameter of 260 nm, a height of 700 nm, and are regularly placed along a 2D array covering a 1 mm × 1 mm^2^ area with a 3 µm spacing. Although this step requires an expensive nanofabrication system, the master structure can then be reused over 20 times to generate a large number of molds and subsequent replicas, spreading the cost of this step across over a hundred devices. The fact that the master structure is only used to fabricate the NIL molds minimizes its potential damage and maximizes its lifetime.

In the second step, a perfluoropolyether (PFPE) mold of the master structure is created to serve as a stamp for the NIL process. This mold is then pressed on a standard microscope borosilicate glass coverslip covered with a thin layer of sol‐gel containing SiO_2_ precursors (Figure 1, step 3).^[^ ^68^, ^69^ ^]^ After thermal curing and solvent evaporation, the nano‐pillar array pattern is transferred onto the microscope coverslip, bearing all the physical properties of silica. During the thermal annealing, a shrinkage of the sol‐gel solution occurs, leading to a reduction of the nanopillar dimensions to a diameter of 230 nm and a height of 400 nm. The 3 µm spacing is unchanged. After the nano‐imprint step 3, a thin residual SiO_2_ layer from the sol‐gel solution covers the zones between the nanopillars. As an optional step of our process (Figure 1, step 4), this residual layer can be removed using reactive ion etching (RIE), ensuring only the silica nanopillars remain on the microscope coverslip substrate. This step ensures better control of the ZMW geometry during the HF etching.

A 150 nm thick opaque aluminium layer is then deposited (Figure 1, step 5). Other metals could be used with our method, yet aluminium is typically preferred to cover a broad spectral range from the UV to the near‐IR and to keep costs low.^[^ ^35^, ^74^ ^]^ During the metal deposition process, a thin layer of aluminium is deposited on the vertical surfaces of the nanopillars, blocking the exposure of the SiO_2_ underneath. To remove these deposits from the sides, the sample is immersed in an acid solution (Aluminium Etchant Type A) that selectively dissolves the aluminium (Figure 1, step 6). With a typical wet etching time of 40 s, the thin aluminium layer on the vertical pillar sides is dissolved, but the film covering the substrate remains sufficiently opaque with a thickness above 100 nm.

In the final step (Figure 1, number 7), all the ZMW apertures are etched simultaneously by selectively dissolving the silica nanopillars using vapor phase HF acid etching in a closed system (Idonus VPE100). In vapor‐phase HF etching, the silica is efficiently etched, but the aluminum bulk remains largely intact.^[^ ^70^, ^75^ ^]^ Vapor‐phase etching is preferred over liquid wet etching as it avoids the re‐deposition issues and is much safer to use. This process creates ZMWs apertures with 230 nm diameter and a clean profile in a massively parallel fashion without requiring expensive milling for each aperture (Figure 1, step 7). The complete fabrication process is completed within 1–2 h and can easily be scaled‐up to wafer‐sized large surfaces once the proper master is fabricated. Top view SEM images corresponding to the different nanofabrication steps are shown in the Figure S1 (Supporting Information). Focused ion beam cross‐cuts of the fabricated ZMWs show complete removal of the SiO2 pillar (Figure S2, Supporting Information). A statistical analysis of the SEM images over a set of 92 ZMWs indicates an average diameter of 230 nm with a standard deviation of 13 nm (5% of the average). The minimum and maximum of the distribution are 200 and 260 nm, respectively (see Figure S3, Supporting Information for details).

To illustrate our capacity to fabricate a large array of ZMW nanoapertures in parallel, we provide optical images of the final structure in Figure S4 (Supporting Information). The array of 330 x 330 ZMWs covers an area of ≈1 x 1 mm^2^. While some local defects due to the NIL process are visible, regular defect‐free arrays of several hundreds of ZMWs can be easily found (Figure S4, Supporting Information). These regions are enough to cover a 150 x 150 µm^2^ field of view of a typical fluorescence microscope equipped with a high numerical aperture objective. Moreover, a small fraction of local defects can be helpful to serve as fiducial marks, enabling the re‐observation of the same zone on the sample.

The choice of the 230 nm diameter results from a trade‐off between nanofabrication complexity and optical performance, based on the characterization performed on FIB‐based fabricated structures.^[^ ^76^, ^77^ ^]^ While the optical confinement increases for smaller diameters, the higher height‐to‐diameter ratio complicates the nanofabrication process. Preliminary results shown in Figure S5 (Supporting Information) indicate that nanopillars with diameters down to 110 nm and a height above 300 nm can be imprinted using our approach, leading to ZMWs with similar diameters. Future work will investigate the properties of these structures, which are still in their development stage. As this work focuses on establishing the nanofabrication concept, we select the nanoapertures with a 230 nm diameter. Additionally, it was reported that smaller ZMW with diameters below 200 nm were leading to a more pronounced observation of nonspecific adsorption of the target fluorescent molecules on the aperture surface.^[^ ^52^ ^]^ Hence, the 230 nm diameter chosen here is an efficient way to circumvent this issue.

**Figure 2:**
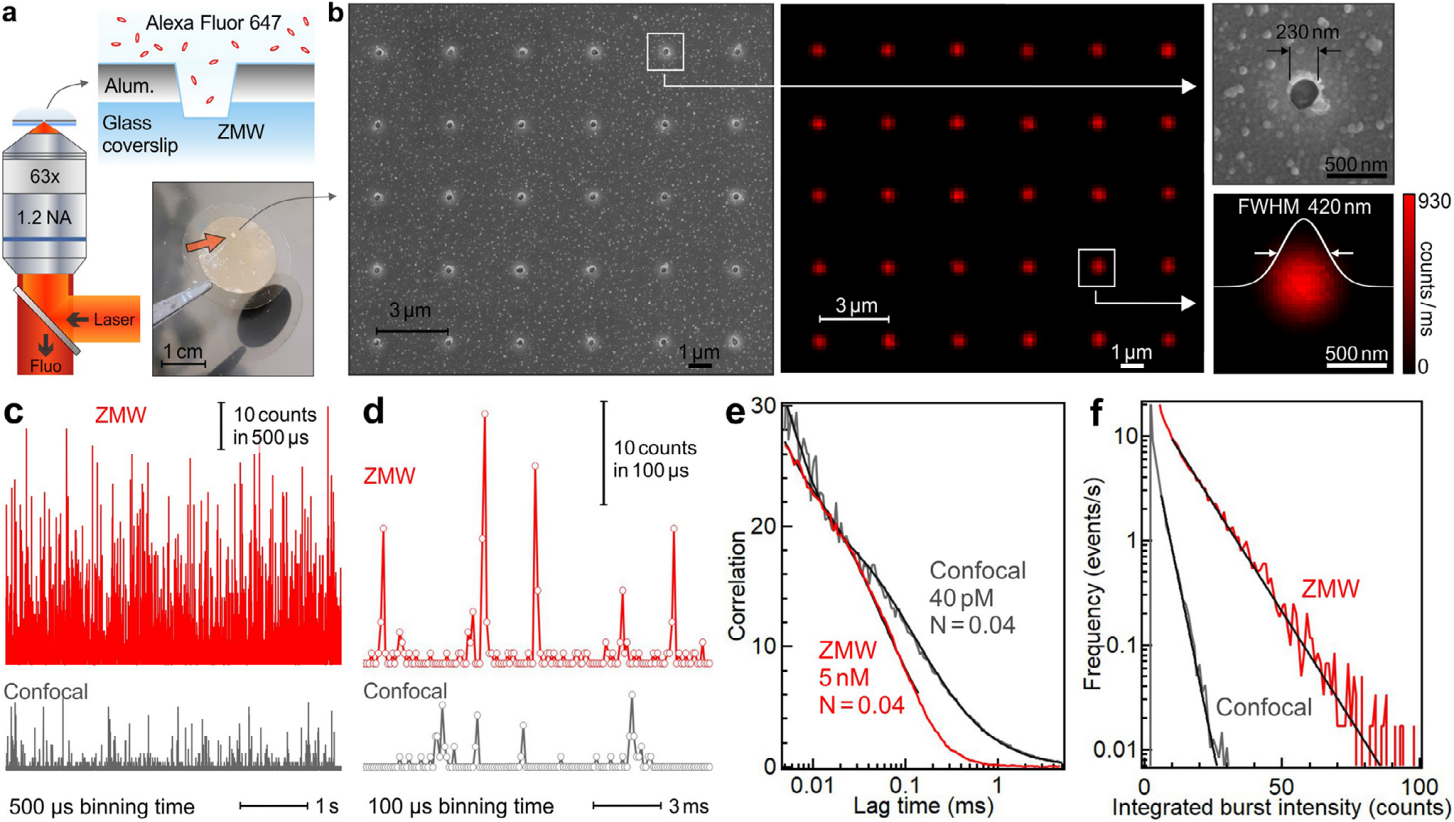
Single‐molecule fluorescence bursts detection in zero‐mode waveguide nanoapertures. a) Scheme of the experimental setup. The insert shows a photograph of the sample (square array marked by an arrow) on a 1 inch glass coverslip. b) SEM and optical confocal fluorescence image of the ZMW array fabricated using the approach in Figure 1. The integration time per pixel in the optical image is 15 ms, the excitation power is 20 µW at 633 nm, and the dye concentration has been increased to 1.4 µm to yield enough signal while scanning. c) Fluorescence time traces for a ZMW and the confocal reference showing typical bursts from diffusing single Alexa Fluor 647 molecules. The binning time is 500 µs, and the laser excitation power is 25 µW. The Alexa 647 concentrations are set so as to have comparable average numbers of molecules detected by FLCS, corresponding to 40 pM for the confocal and 5 nm for the ZMW. d) Close‐up views of fluorescence bursts from (c) with a 100 µs binning time, showing the possibility to reach high temporal resolution while maintaining a high signal with the ZMW. Supplementary time traces are shown in Figure S6 (Supporting Information). e) FLCS correlation data corresponding to the time traces in (c) show a similar amplitude and a similar average number of molecules in each detection volume. f) Histograms of the integrated burst intensities for both traces. The black lines are single exponential fits to the data.

**Figure 3:**
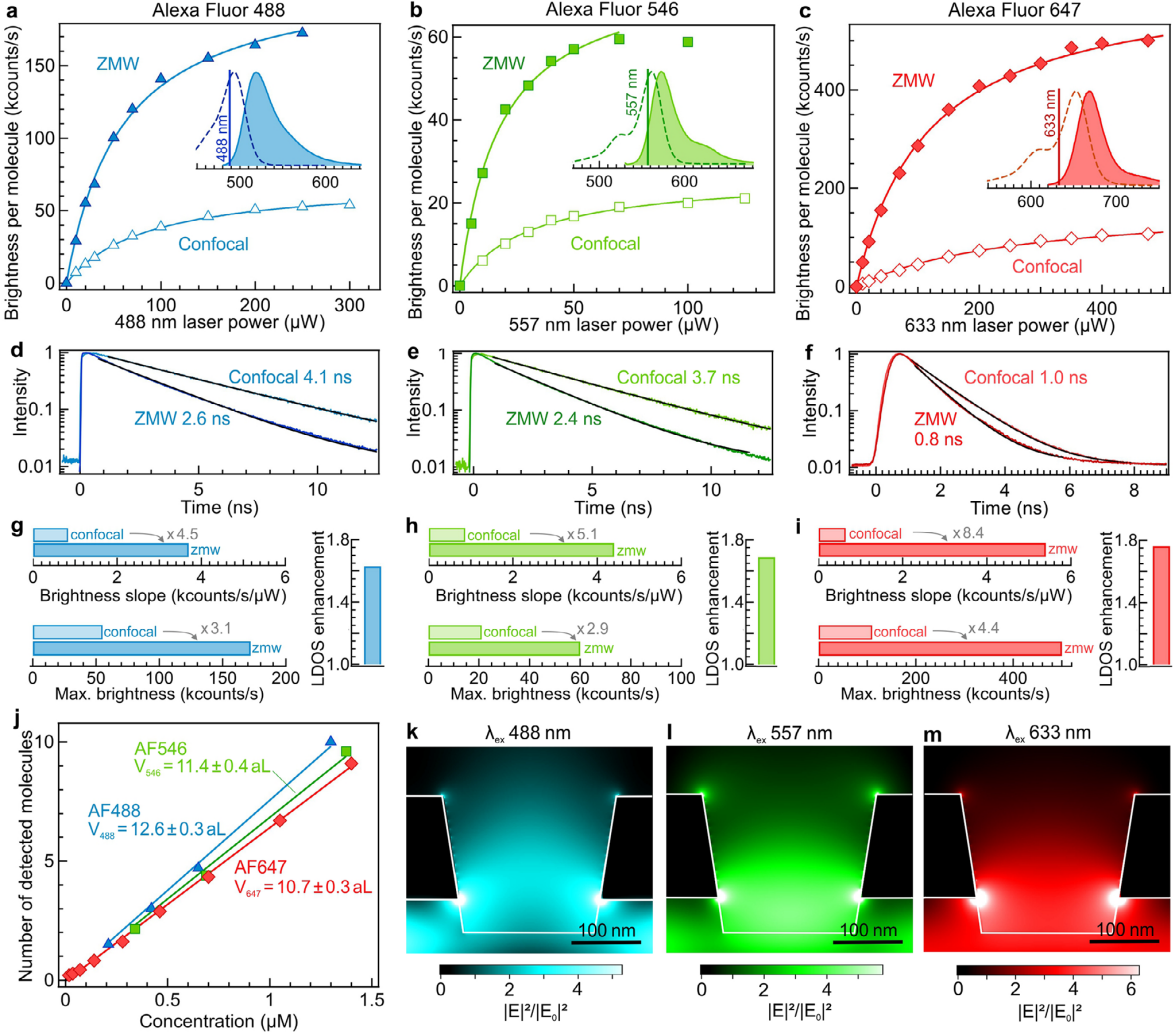
Multicolor operation of ZMW apertures to enhance single‐molecule fluorescence. a–c) Fluorescence brightness per molecule *CRM* as a function of the laser excitation power *P* for Alexa Fluor 488 (a), Alexa Fluor 546 (b), and Alexa Fluor 647 (c). Lines are numerical fits using the model A*P/(1 + *P*/*P_sat_*) where *A* is the slope in the linear low‐excitation regime and is the saturation power *P_sat_*. The absorption (dashed) and emission (solid filled) spectra are displayed in the insert, with the laser lines at 488, 557, and 635 nm indicated by a vertical bar. d–f) Normalized fluorescence lifetime decays corresponding to the different cases in (a–c). Black lines are numerical fits using a single exponential decay. g–i) Comparison of the brightness slope *A*, the saturation power *P_sat_* and the LDOS enhancement for the different dyes deduced from the data in (a‐f). For the experiments in (a‐i), the dye concentrations were 1.4 µm for the ZMWs and 20 nm for the confocal cases. j) Number of molecules *N* detected by FCS as a function of the dye concentration. The raw FCS data are presented in Figure S11 (Supporting Information). Lines are numerical fits used to determine the effective detection volume for each excitation wavelength. The error bars correspond to one standard deviation. k–m) Finite‐difference time‐domain simulations of the excitation intensity enhancement inside the ZMW for different laser wavelengths. The light is coming from the bottom of the ZMW.

To characterize the optical performance of the fabricated ZMW apertures, we record the fluorescence emission of Alexa Fluor 647 dyes diffusing in PBS solution (**Figure** **2**a,b). The purpose of this experiment is to highlight the capability to detect single fluorescent molecules as they diffuse across the detection volume and emit fluorescence bursts. A more detailed quantification of the emission properties for different Alexa Fluor dyes is discussed later in **Figure** **3**. Apart from the ZMW replacing the usual microscope glass coverslip, the rest of the microscope system is a standard confocal setup (see details in the Experimental Section section). In order to demonstrate our ability to detect single diffusing molecules, we start with highly diluted conditions (5 nm for the ZMW, 40 pM for the confocal reference) so that, on average 0.04 molecules are present in the respective detection volume of each setup (Figure 2c,d). As the number of emitters is low, we use here fluorescence lifetime correlation spectroscopy (FLCS) to yield a more accurate measurement of the average number of molecules for each experiment, using the fluorescence lifetime information to improve the discrimination of the signal against the background (Figure 2e).^[^ ^78^, ^79^, ^80^ ^]^ The fluorescence time traces in Figure 2c,d, and the longer traces in Figure S6 (Supporting Information) clearly show emission bursts from single diffusing molecules, with a peak intensity 5× higher for the ZMW as compared to the confocal reference (averaged over the 500 brightest bursts with 500 µs binning time), demonstrating single molecule fluorescence enhancement. Even if the fluorescence intensity is integrated over the total burst duration, the ZMW remains ≈4× brighter (Figure 2f). This intense emission allows for reducing the binning time for the time trace and increasing the temporal resolution. Down to 100 µs, where the peaks become hard to distinguish on the confocal setup, the ZMW‐enhanced signal enables a significant improvement in the burst resolution (Figure 2d; Figure S3d, Supporting Information). Another advantage of ZMWs is that the detection rate (number of bursts per second) is higher as a consequence of the reduced diffusion time and the higher signal, enabling better discrimination of the bursts against the background.^[^ ^15^ ^]^ This allows for a significant reduction in the integration time needed to accumulate a given number of fluorescence bursts, with a gain of over 2 orders of magnitude as compared to the confocal case (Figure S7, Supporting Information).

We characterize the optical performance of the ZMW apertures for three different fluorescent dyes (Alexa Fluor 488, 546, and 647) at three different laser excitation wavelengths (488, 557, and 633 nm). These Alexa Fluor dyes are selected to cover a broad spectral range and because they have been demonstrated to have minimal affinity for the glass and alumina surfaces, allowing to use of the ZMWs without supplementary surface passivation steps.^[^ ^52^ ^]^ Fluorescence correlation spectroscopy (FCS) is used to determine the average number of molecules for each experiment and their fluorescence brightness per emitter (*CRM* count rate per molecule),^[^ ^81^ ^]^ in a similar fashion to our earlier works on ZMWs fabricated by FIB.^[^ ^15^, ^30^, ^74^, ^76^ ^]^ The results of the brightness *CRM* for the 3 different dyes are summarized on **Figure** **3**a–c as function of the excitation power. We also record the fluorescence lifetime decay traces, observing a reduction of the fluorescence lifetime for all cases.^[^ ^30^, ^82^ ^]^

From this experimental dataset, we extract different quantities (Figure 3g–i): i) the slope of the brightness as function of the excitation power, representing the emission in the linear excitation regime, ii) the maximum detected count rate, corresponding to the emission in the saturation regime, and iii) the enhancement of the local density of optical states (LDOS).^[^ ^12^ ^]^ For each case, the enhancement as compared to the confocal reference is noted. In the linear excitation regime, the brightness enhancement factors are 4.5× for Alexa Fluor 488, 5.1× for Alexa Fluor 546, and 8.4× for Alexa Fluor 647, demonstrating a significant gain enabled by the ZMW across a broad spectral range. The fact that the enhancement is higher for Alexa Fluor 647 than for Alexa Fluor 488 is a consequence of the lower quantum yield of Alexa Fluor 647 (33%) as compared to Alexa Fluor 488 (92%). Supplementary contributions stem from the lower plasmonic losses of aluminium in the red region and the fact that longer wavelengths in the red lead to a better optical confinement inside the ZMW.^[^ ^77^ ^]^ In the saturation regime, the higher excitation intensity inside the ZMW no longer contributes to the detected signal, and hence the apparent gains are lower. Nevertheless, we still obtain 3.1× for Alexa Fluor 488, 2.9× for Alexa Fluor 546, and 4.4× for Alexa Fluor 647, showing the ZMW ability to enhance the net detected signal across a broad range of conditions.^[^ ^31^ ^]^ The LDOS enhancement represents the ability of the ZMW aperture to accelerate the dye photodynamics.^[^ ^12^, ^15^, ^30^ ^]^ This quantity is computed by correcting the observed fluorescence lifetime reduction for the contribution of the intrinsic nonradiative decay rate of each dye using their calibrated reference quantum yields (see details in Experimental Section) so as to keep only the effect of the photonic environment (enhanced emission rate and ohmic losses into the metal). Again, we observe a slight increase in the LDOS enhancement from 1.6 ± 0.1 for Alexa Fluor 488 to 1.8 ± 0.1 for Alexa Fluor 647, which we relate to the lower plasmonic losses and the better optical confinement in the red part of the spectrum.

The ability to measure the number of molecules is demonstrated in Figure 3j through a series of FCS experiments on dilutions from a solution of calibrated concentration. The slope of the linear dependence gives the ZMW detection volume, typically in the 10 aL (10^−18^ L) range that corresponds to a 100× reduction as compared to a 1 fL confocal detection volume. These ZMW volumes determined experimentally correspond well to the intensity distributions computed numerically (Figure 3k–m), which indicate propagation lengths (from the glass‐water interface, including a 50 nm undercut into the glass substrate) of 250, 225, and 200 nm for the laser wavelengths of 488, 557, and 633 nm. Additional experimental results shown in the Supporting Information detail the influence of the HF etching time on the ZMW performance (Figure S8, Supporting Information), revealing an increase in the detection volume for longer etching times. A statistical analysis of the FCS results indicates that the experimental uncertainties correspond to 10% for *N* and *CRM*, and 15% for τ_*d*_ (Figure S9, Supporting Information). No statistical difference in the data was found over four re‐uses of the ZMW sample (Figure S10, Supporting Information), demonstrating appropriate re‐usability of the devices. Altogether, the experimental results in Figure 3 and Figures S8–S11 (Supporting Information) demonstrate the ability of the ZMW to confine light on a sub‐wavelength scale, leading to enhanced fluorescence brightness and accelerated emission dynamics for a broad spectral range covering nearly all the visible spectrum.

**Figure 4:**
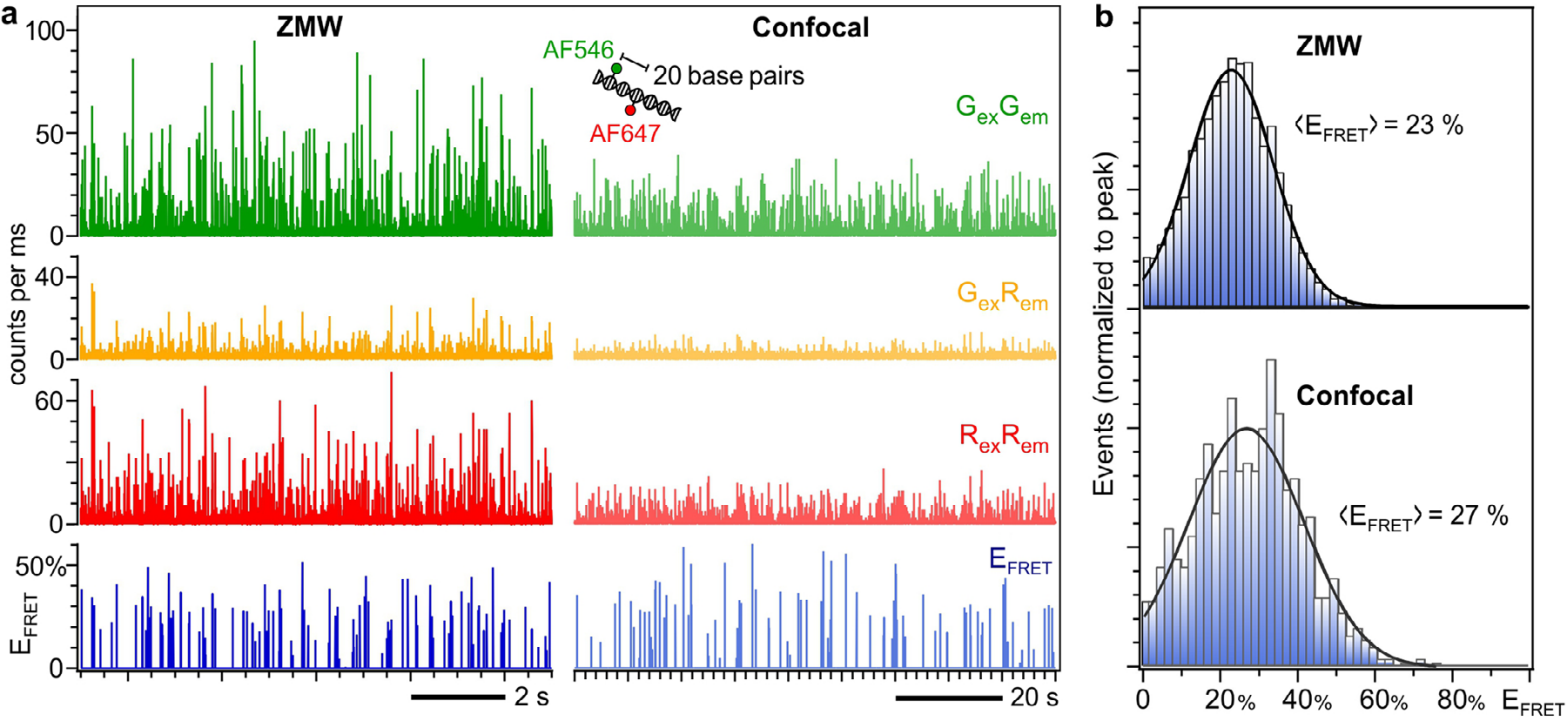
Single‐molecule FRET data in ZMW nanoapertures. a) Fluorescent time traces of single Alexa546‐Alexa647 FRET pairs diffusing in the ZMW (left) and the confocal setup (right). G_ex_G_em_ denotes the donor emission after donor direct excitation at 557 nm, G_ex_R_em_ is the FRET emission (acceptor fluorescence) after donor excitation at 557 nm, and R_ex_R_em_ is the acceptor emission after acceptor direct excitation at 635 nm. The bottom trace is the FRET efficiency computed according to Equation (4). The binning time is 1 ms. The DNA concentrations are 6 nm for the ZMW and 40 pM for the confocal, ensuring a similar average number of molecules of 0.04 ± 0.01 for both cases. b) smFRET efficiency histograms measured in the ZMW and the confocal setup. Black lines are numerical fits with a Gaussian distribution to determine the average FRET efficiency indicated on the graph. The total integration time for the ZMW is 6 mins (4430 bursts detected), while 20 min were used for the confocal reference (1220 bursts detected).

We take advantage of the broad spectral operability of the ZMW apertures to demonstrate the ability to perform a single‐molecule Förster resonance energy transfer (smFRET) experiment (**Figure** **4**).^[^ ^5^, ^83^ ^]^ Here we use Alexa Fluor 546 donor and Alexa Fluor 647 acceptor dyes separated by 20 base pairs on a double‐stranded DNA molecule. Pulsed interleaved excitation (PIE) and post‐selection are used to select only the molecules bearing both fluorescent donor and acceptor molecules.^[^ ^84^, ^85^ ^]^ The analysis protocol follows the guidelines agreed in this field (see Experimental Section for details).^[^ ^83^ ^]^ The motivation here is not to investigate in detail the FRET photophysics in ZMWs, which was partly addressed in some of our previous works^,[^ ^74^, ^77^, ^86^ ^]^ but instead to demonstrate the applicability of the NIL‐fabricated ZMWs in a smFRET context. Figure 4a shows typical fluorescence time traces recorded with the ZMW and the confocal setup, with fluorescence bursts stemming from single diffusing FRET pairs. As for Figure 2, we observe a clearly higher brightness in the ZMW as compared to the confocal reference. This gain is especially critical for the red emission events after green excitation (G_ex_R_em_, Figure 4a), which contain the relevant FRET signal. The FRET efficiency E_FRET_ histograms (Figure 4b) are then computed from the time traces,^[^ ^83^ ^]^ taking into account the donor crosstalk into the acceptor channel, the direct excitation of the acceptor dye by the green laser, the differences in quantum yields, and the detection efficiencies of the separate detection channels (see details in the Experimental Section section). In the presence of the ZMW, we also take into account the different brightness enhancement factors between the two dyes.^[^ ^74^, ^86^ ^]^ The average FRET efficiency is slightly reduced for the ZMW as compared to the confocal reference (22.9 ± 0.2 % for the ZMW versus 26.8 ± 0.6 % for the confocal reference; the uncertainty is the statistical error on determining the average value, systematic errors in the computation of E_FRET_ are not included). This reduction is related to the ohmic losses into the metal and the higher LDOS inside the ZMW, which drives the radiative emission as compared to the near‐field energy transfer route, and was observed previously on similar FRET constructs.^[^ ^31^, ^77^ ^]^ This result does not contradict the enhancement of the FRET process for longer donor‐acceptor separations reported in Refs.[74, 86], as these works already pointed out that a separation larger than 10 nm (≈30 base pairs) was needed in order to observe the FRET efficiency enhancement. Another interesting aspect of the ZMW use is the reduction in the width of the FRET histogram,^[^ ^74^ ^]^ which directly improves the accuracy of the smFRET observations. We find a standard deviation of 11% for the ZMW as compared to 15% for the confocal reference. This effect is explained by the higher fluorescence brightness in the ZMW, which reduces the shot noise contribution,^[^ ^87^, ^88^ ^]^ and to a lesser extent by the higher number of detected events, reducing the sampling noise (Figure S7, Supporting Information).

## 3 Conclusion

We have described a novel methodology to fabricate ZMW arrays of high optical performance in a simple, cost‐effective, and scalable approach. The key innovation relies on the combination of sol‐gel nanoimprint lithography with hydrofluoric acid vapor etching for high‐throughput sample fabrication. The high optical performance of the resulting ZMW arrays is demonstrated through a series of single‐molecule experiments using fluorescence burst analysis, FCS, and smFRET. For a broad range of fluorescent dyes covering nearly all the visible spectrum, we achieve attoliter detection volumes 100× below the confocal diffraction limit, together with enhanced fluorescence brightness and accelerated photodynamics. We show that the operation of ZMWs is straightforward in confocal single‐molecule fluorescence detection, enabling improved signal‐to‐noise ratios, better temporal resolution, faster data acquisition, and operation at micromolar concentrations.

This nanofabrication strategy represents a significant step forward in bridging the technological gap between nanophotonics and single‐molecule biophysics.^[^ ^49^ ^]^ Enabling broader access to ZMW technology opens new opportunities to advance biophysics and biosensing applications well beyond the current limits of confocal or total internal reflectance (TIRF) microscopy.^[^ ^29^, ^39^, ^40^, ^41^, ^42^, ^43^, ^44^, ^45^, ^46^, ^47^, ^48^ ^]^ The scalability of this approach supports the fabrication of more complex 3D nanostructures at moderate cost, for instance, to include a microreflector enabling a supplementary increase in the fluorescence collection efficiency.^[^ ^15^ ^]^ Beyond single‐molecule detection, ZMW nanoaperture arrays are also relevant for applications in plasmonics biosensing,^[^ ^71^, ^72^, ^73^ ^]^ nanophotonic filters,^[^ ^23^ ^]^ microscope calibration,^[^ ^89^ ^]^ and nanoscale surface patterning.^[^ ^90^ ^]^ The final ZMWs are fabricated on standard borosilicate glass coverslips, allowing direct compatibility with common silane‐based surface functionalization methods.^[^ ^90^, ^91^ ^]^ Altogether, this work establishes a practical and powerful platform for realizing aperture‐based nanophotonics devices. By lowering the technological barrier to ZMW fabrication, this work paves the way for broader adoption of advanced nanophotonics tools in both fundamental research and applied biosensing technologies

## 4 Experimental Section

### ZMW Sample Fabrication

The ZMW nanoapertures were fabricated by combining nanoimprint lithography (NIL) and selective vapor phase etching (VPE) of silicon dioxide versus aluminum. The process starts with the fabrication of a nanostructured silicon master consisting of nano‐pillars, obtained by electron beam lithography (EBL) using a Raith Pioneer system equipped with a field‐emission electron gun (FEG). The nano‐pillar array covers a 1 mm × 1 mm^2^ area patterned on a silicon (100) substrate. The nano‐pillars were fabricated using a top‐down approach, with a diameter of 260 ± 10 nm, a height of 700 ± 10 nm, and a spacing of 3 µm. For the lithography step, a positive‐tone resist was spin‐coated onto the substrate and exposed to an electron beam.^[^ ^92^ ^]^ After development, a thin layer of pure nickel was evaporated onto the patterned sample and then lifted off, forming a hard mask for reactive ion etching. The nickel mask exhibits excellent resistance to fluorine plasma,^[^ ^92^ ^]^ which was required for etching vertical silicon pillars using a sequential SF_6_/O_2_ multistep RIE process that alternates between etching and passivation steps. Finally, following the RIE step, the remaining nickel mask was removed from the etched pillars using a wet chemical bath, and the etching depth (equal to the pillar height) was measured with a contact profilometer.^[^ ^92^ ^]^ The silicon master structure was stored in a clean plastic box at room temperature and in the dark.

Molds in perfluoropolyether (PFPE) were then made from the master after a hydrophobic surface treatment using a solution of perfluorinated trichlorosilane. The same master can be used to produce multiple molds. The pattern was transferred onto cleaned borosilicate glass coverslips (diameter 1 inch; thickness 150 µm) via NIL using a sol‐gel solution containing tetraethyl orthosilicate (TEOS) as SiO_2_ precursors (Solnil solution C). The deposition prior to the nanoimprint was carried out by spin coating at 4000 rpm for 10 s, followed by the application of the mold. The assembly (mold and coverslip) was then heated at 70 °C for 2 min to promote solvent evaporation and pattern stabilization. After demolding, the imprinted patterns were thermally annealed at 500 °C for 30 min, which leads the imprinted patterns to bear all the physico‐chemical properties of fused silica.

A reactive ion etching (RIE) step was performed for 1 min to remove the residual SiO_2_ layer between the nano‐pillars, thus ensuring selective etching in the subsequent steps. RIE etching was performed using a Plassys MG 200 system. The process employed flow rates of 20 sccm SF_6_ and 8 sccm O_2_ at a pressure of 22 mTorr with a power of 120 W supplied by an RF generator at 13.56 MHz.

A 150 nm Al thin film was deposited at room temperature by electron‐beam physical vapor deposition (EBPVD, Bühler Syrus Pro 710) using a high‐purity (99.99%) Al target and a deposition rate of 10 nm.s^−1^. The deposition chamber was maintained under vacuum at ≈1 × 10^−7^ mbar, with the substrate holder rotating at 30 rpm to ensure uniform film thickness. The residual aluminum deposited around the nano‐pillars was then partially removed by wet etching, using “Aluminum Etchant Type A” (Sigma–Aldrich), a mixture of phosphoric acid (>77%), nitric acid (<3%), and acetic acid (<3.5%), for 40 s before rinsing with MilliQ water and drying with nitrogen. The selective etching of silica nano‐pillars was then carried out by HF vapor‐phase etching for 50 s using a VPE100 system (Idonus), with an evaporation temperature maintained at 35 °C using a 10% liquid HF solution.

The morphological analysis of the samples was carried out using scanning electron microscopy (SEM) with a FEI DualBeam DB235 Strata. Cross‐sections of the nanoapertures were obtained using a gallium‐based focused ion beam (FIB) at 30 keV and 100 pA.

### Fluorescent Molecule Samples

Carboxylate‐modified Alexa Fluor dyes were purchased from ThermoFisher and used as received. All experiments were performed in standard 1x Phosphate‐Buffered Saline (PBS) solution containing 140 mM NaCl, 10 mM phosphate buffer, and 3 mM KCl, pH 7.4 at 25 °C. The FRET sample consists of double‐stranded DNA with the forward strand being labelled with Alexa Fluor 546 (donor) and its complementary strand with Alexa Fluor 647 (acceptor). The DNA strands were obtained from IBA Life Solution (Göttingen, Germany) and were HPLC purified. The forward strand sequence of the DNA was 5′‐CCT GAG CGT ACT GCA GGA TAG CCT ATC GCG TGT CAT ATG CTG TTC AGT GCG‐3′, where the thymine at position 44 was labelled with Alexa Fluor 546. The complementary reverse strand was 5′‐CGC ACT GAA CAG CAT ATG ACA CGC GAT AGG CTA TCC TGC AGT ACG CTC AGG‐3′, where the T base at position 27 was labelled with Alexa Fluor 647. In this configuration, a 20 base pairs separation between donor and acceptor dyes was obtained, corresponding to ≈8.7 nm, assuming the linker distance between the DNA and the emitting center was 1.5 nm. The forward and reverse strands were hybridized in a buffer containing 5 mM Tris, 20 mM MgCl_2_, 5 mM NaCl at pH 7.5, with the solution being heated at 90 °C for 5 min before slow cool down to room temperature in 2.5 h. The smFRET experiments were performed in a buffer containing 20 mM Hepes, 10 mM NaCl, 0.1% Tween 20 at pH 7.5.^[^ ^74^, ^86^ ^]^

### Optical Microscope

All the optical characterization experiments were performed using a custom‐built confocal microscope (Nikon Ti‐U Eclipse), already described in earlier studies.^[^ ^15^, ^74^ ^]^ Briefly, the microscope was equipped with different laser lines at 488 nm (continuous, Coherent Sapphire), 490 and 557 nm (pulsed 3 ps 40 MHz, Toptica iChrome TVIS), 633 nm (continuous, Melles Griot HeNe), and 635 nm (pulsed 70 ps 40 MHz, Picoquant LDH). All the laser beams pass through the same polarization‐maintaining single mode optical fiber (Thorlabs), defining a similar beam profile at the fiber output before the entrance in the back port of the microscope. For the smFRET experiments, 557 and 635 nm laser pulses were alternating at 40 MHz repetition rate in a pulsed interleaved excitation (PIE) configuration,^[^ ^84^, ^85^ ^]^ with 20 µW average power low enough to avoid fluorescence saturation or photobleaching on the diffusing dyes. All lasers have linear polarizations that were set parallel to each other. No polarization selection was performed on the fluorescence detection. A multiband dichroic mirror (ZT 405/488/561/640rpc, Chroma) reflects the laser beam toward the microscope. The molecules were excited using a 63x, 1.2 NA water immersion (Zeiss C‐Apochromat), and the emission was collected by the same objective in epifluorescence configuration. To reject the backscattered laser light, the emission was passed through the same multiband dichroic mirror and a multi‐band through emission filter (ZET405/488/565/640mv2, Chroma). 50 µm confocal pinholes were used for all the experiments. The green detection channel was equipped with an MPD‐5CTC single photon avalanche photodiode (Picoquant), while the red detection channel uses an SPCM AQR 13 (Perkin Elmer). Bandpass filters were used in front of each avalanche photodiode corresponding to 500–560 nm for Alexa Fluor 488, 570–620 nm for Alexa Fluor 546 (ET570LP and ET595/50m, Chroma), and 650–750 nm for Alexa Fluor 647 (ET655LP, Chroma). For smFRET experiments, the ET655LP filter was replaced with a bandpass filter (670DF40, Omega) to better reject the Raman scattered signal excited by the 557 nm laser. The photodiode signals were connected to a HydraHarp400 single photon counting module (Picoquant) in a time‐tagged time‐resolved (TTTR) mode. Fluorescence lifetime measurements have a temporal resolution of 38 ps upon green excitation (490 and 557 nm) and 400 ps upon red excitation (635 nm) defined as the full width half maximum of the instrument response function.

### Fluorescence Correlation Spectroscopy

All the analysis was performed using Symphotime64 software (Picoquant). The FCS curves were fitted using the following equation, where we use a 3D Brownian diffusion model with an additional term to include the blinking effects.^[^ ^52^, ^81^, ^93^ ^]^

(1) $$G\left(\tau \right)=\rho \left[1+\frac{T_{ds}}{1-T_{ds}}\exp\left(-\frac{\tau }{\tau_{ds}}\right)\right]{\left(1+\frac{\tau }{\tau_{d}}\right)}^{-1}{\left(1+\frac{1\;\tau }{s^{2}\tau_{d}}\right)}^{-0.5}\;$$

where *G*(τ) is the correlation amplitude, ρ is the correlation amplitude, *T_ds_* is the fraction of molecules going in the dark state, τ_ds_ is the blinking time of the dark state, τ_d_ is the mean diffusion time, and *s* corresponds to the aspect ratio of the axial to the transversal dimension of the excitation source in the solution. For the confocal case, we used a value of *s* = 5, whereas for the optical horn antennas, based on our past results for the ZMW aperture,^[^ ^30^, ^76^ ^]^ we used a value of *s* = 1 which fits well the experimental correlation amplitude. To extract the fitting parameters such as the number of molecules and the diffusion time, for the confocal case, we fit the experimental data from 3 µs to 10 ms lag time, whereas for the ZMW and horn antenna, the data was fitted from 3 µs to 0.1 ms, as the fitting model starts to divert from the experimental data at longer lag times. From the fitting values of the correlation amplitude ρ, the measured background *B* on the ZMW, and the total detected intensity *F*, we extract the average number of molecules *N* and the brightness or count rate per molecule *CRM* as:^[^ ^15^ ^]^

(2) $$N=\frac{1}{\rho }{\left(1-\frac{B}{F}\right)}^{2}$$

(3) $$CRM=\frac{1}{N}\left(F-B\right)$$

*N* corresponds to the average number of detected fluorescent molecules in the observation volume over the duration of the experiment.^[^ ^81^ ^]^ Alternatively, the fluorescence lifetime information can be used to compute a filter function based on the TCSPC histogram. This filter function can, in turn, be used to compute the lifetime‐filtered intensity correlation function for FLCS, in a similar fashion as used in our recent work.^[^ ^93^ ^]^ Both background‐corrected FCS and FLCS yield similar results in the case of this study.

The LDOS enhancement is computed as $LDOS_{enh}=1/\phi_{0}\left(\tau_{LT}^{0}/\tau_{LT}^{*}-1+\phi_{0}\right)$ where ϕ_0_ is the reference quantum yield of the fluorescent dye in a water solution, $\tau_{LT}^{0}$ and $\tau_{LT}^{*}$ are the fluorescence lifetimes in the confocal setup and the ZMW, respectively.^[^ ^15^, ^30^ ^]^ The rationale behind this formula was to correct the influence of the intrinsic nonradiative decay rate constant on the observed fluorescence lifetime in order to measure only the optical influence of the ZMW. Note that the nonradiative losses in the metal are taken into account in this formula, as they are induced by the optical environment.^[^ ^15^, ^30^ ^]^ The reference quantum yields ϕ_0_ are taken from tabulated data to be 92%, 79% and 33% for Alexa Fluor 488, 546 and 647 respectively.

### FRET Analysis

The FRET efficiency histograms were also computed using Symphotime64 software (Picoquant) following a standard PIE approach in smFRET.^[^ ^83^, ^85^ ^]^ First, the single molecule detection events were selected, and apply a threshold criterion above the noise level. A second threshold was used to check the presence of the red dye upon excitation by the red laser.^[^ ^74^ ^]^ The FRET efficiency is calculated as^[^ ^74^ ^]^

(4) $$E_{FRET}=\frac{n_{A}^{green}-\alpha n_{D}^{green}-\delta n_{A}^{red}}{\left(n_{A}^{green}-\alpha n_{D}^{green}-\delta n_{A}^{red}\right)+\gamma n_{D}^{green}}$$

where $n_{D}^{green}$ and $n_{A}^{green}$ the number of photons in each burst for the donor and acceptor channel upon excitation by a green laser, $n_{A}^{red}$ number of photons in the red channel with the excitation by the red laser. The numbers of photons were corrected for the background contribution in each channel. The background counts were measured by performing a separate experiment using the buffer solution only for the ZMW or the reference glass coverslip. The crosstalk α is the ratio between the donor emission leaking into the acceptor channel as compared to the donor emission in the donor channel. The crosstalk is determined for a DNA sample containing only the donor fluorophore: $\alpha =\frac{n_{A}^{green}}{n_{D}^{green}}$ with α = 0.1 for our dyes, with no detectable difference between the ZMW and the confocal setup. The direct excitation δ corresponds to the fraction of the acceptor fluorescence due to direct excitation by the green laser, as compared to the acceptor emission signal upon the red laser. This parameter was measured using a DNA sample containing only the acceptor dye: δ $=\frac{n_{A}^{green}}{n_{A}^{red}}$ with δ = 0.1, again with no detectable difference between the ZMW and the confocal setup. The γ correction factor takes into account the differences in the fluorescence quantum yields (φ_d_, φ_a_) and the detection efficiencies (κ_d_, κ_a_): $\gamma =\frac{\kappa_{A}\phi_{A}}{\kappa_{D}\phi_{D}}$. For the Alexa 546‐Alexa647 FRET pair in our confocal setup, a value of γ_conf_ = 0.57 ± 0.02 was computed from the fluorescence spectrum and quantum yield of each dye. As noted in our earlier works on smFRET in ZMWs,^[^ ^74^, ^86^ ^]^ γ is modified in the presence of the ZMW due to the different enhancement factors of the donor and acceptor dyes:^[^ ^74^, ^86^ ^]^ $\gamma_{ZMW}=\gamma_{conf}\times EnhCRM_{AO}^{red}\;/\;EnhCRM_{DO}^{green}$ where $EnhCRM_{AO}^{red}$, $EnhCRM_{DO}^{green}$ are the fluorescence brightness enhancement factors for the acceptor‐only and donor‐only samples. A slight increase in the γ correction factor in the ZMW is found compared to the confocal reference (γ_ZMW_∼0.78 while γ_conf_ = 0.57).

### Numerical Simulations

The electric field intensity maps shown in Figure 3k–m are obtained using with finite‐difference time‐domain (FDTD) method using RSoft Fullwave software. The ZMW geometry was set according to the experimental parameters deduced from the SEM images. The undercut depth into the glass substrate was set to 50 nm. The aluminum complex refractive index was taken from ref. [94]. The borosilicate glass substrate has a refractive index of 1.52, with the ZMW inner volume and top space being filled with water (refractive index 1.33). The simulations use a 2 nm mesh size and were checked for convergence after over 8 optical periods.

### Statistical Analysis

Pre‐processing: for the PIE‐FRET experiments, the intensity time traces were filtered by selecting the pulses where both the donor and acceptor molecules were present and fluorescent active, as commonly performed in this field.^[^ ^83^, ^85^ ^]^ No other processing or filtering was applied for the FCS experiments. The data presentation corresponds to the average value, with the error bars corresponding to the standard deviation. The sample size for each statistical analysis was detailed in the corresponding figure legend. Statistical tests were not relevant to this study and were not used. The statistical analysis was performed using Symphotime64 (Picoquant) and IgorPro 7 (Wavemetrics).

## Conflict of Interest

The authors A.G. and B.K. are employees of the Solnil company. D.G. is a co‐founder of the Solnil company. The remaining authors declare no conflict of interest.

## Supporting information



Supporting Information

## Data Availability

The data that support the findings of this study are available from the corresponding author upon reasonable request.
