# Supplementary material for: High‐Throughput Fabrication of Zero‐Mode Waveguide Nanoaperture Arrays with Sol‐Gel Nanoimprint Lithography for Enhanced Single Molecule Fluorescence Detection
Source: Small. 2025 Nov 21;22(2):e10587. doi: 10.1002/smll.202510587 (PMC12781627; doi:10.1002/smll.202510587)
Supplement: Supplementary file 1 — Supporting Information [file SMLL-22-e10587-s001.pdf]

NANO · MICRO
small

Supporting Information

for *Small*, DOI 10.1002/smll.202510587

High-Throughput Fabrication of Zero-Mode Waveguide Nanoaperture Arrays with Sol-Gel Nanoimprint Lithography for Enhanced Single Molecule Fluorescence Detection

*Hamza Khelidj, Anthony Gourdin, Igor Ozerov, Antonin Moreau, Badre Kerzabi, David Grosso and Jérôme Wenger**

Supplementary Information for

High-Throughput Fabrication of Zero-Mode Waveguide Nanoaperture Arrays with Sol-Gel Nanoimprint Lithography for Enhanced Single Molecule Fluorescence Detection

Hamza Khelidj,[1] Anthony Gourdin,[2] Igor Ozerov,[3] Antonin Moreau,[1] Badre Kerzabi,[2] David Grosso,[2,3] and Jérôme Wenger[1,*]

[1] Aix Marseille Univ, CNRS, Centrale Med, Institut Fresnel, AMUTech, 13013 Marseille, France

[2] Solnil, 163 Avenue de Luminy, 13009 Marseille, France

[3] Aix-Marseille Univ, CNRS, CINaM, Campus de Luminy 13288, Marseille, France

** Corresponding author: jerome.wenger@fresnel.fr*

Contents:

S1. Top view SEM images after different nanofabrication steps

S2. Cross-cut views of single ZMW apertures

S3. Statistical analysis of ZMW diameter

S4. Optical image of the ZMW array

S5. Nanoimprint of pillars with smaller diameters

S6. Fluorescence time traces showing single molecule bursts

S7. Total integration time required to record 1000 single molecule bursts

S8. Influence of HF etching time

S9. Statistical dispersion of the data

S10. Re-usability of the sample

S11. Raw FCS data for different dyes

S1. Top view SEM images after different nanofabrication steps

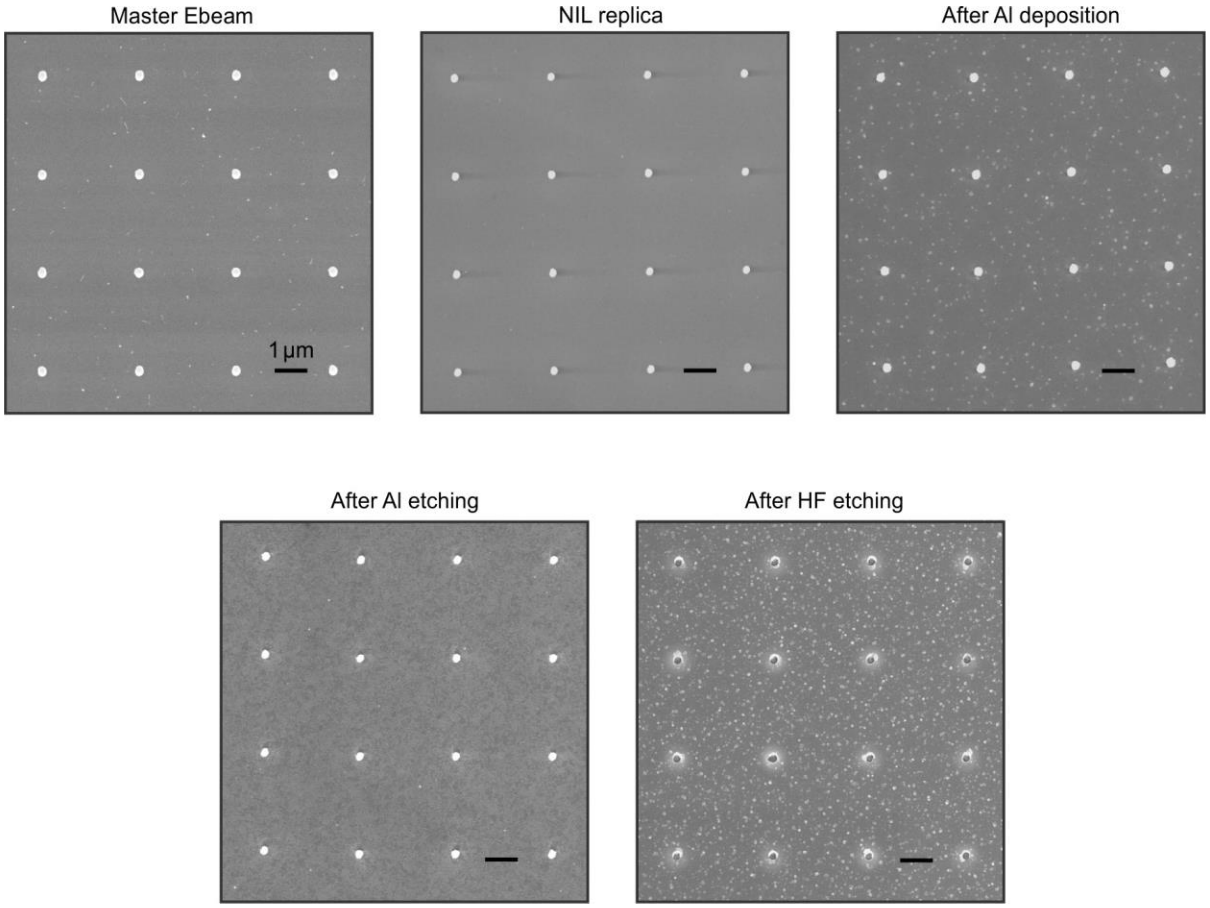

Figure S1. Top view SEM images corresponding to the different nanofabrication steps depicted in Fig. 1. All the scale bars denote 1 µm length.

S2. Cross-cut views of single ZMW apertures

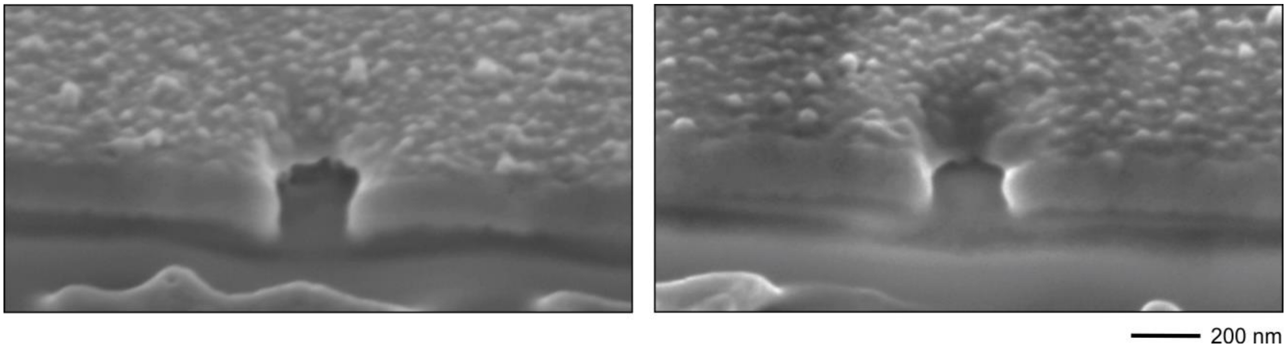

Figure S2. Cross-cut views of single ZMW apertures fabricated using the approach in Fig. 1. Half of the sample has been cut by focused ion beam (FIB) milling. The sample surface is tilted by 52° respective to the SEM imaging direction.

S3. Statistical analysis of ZMW diameter

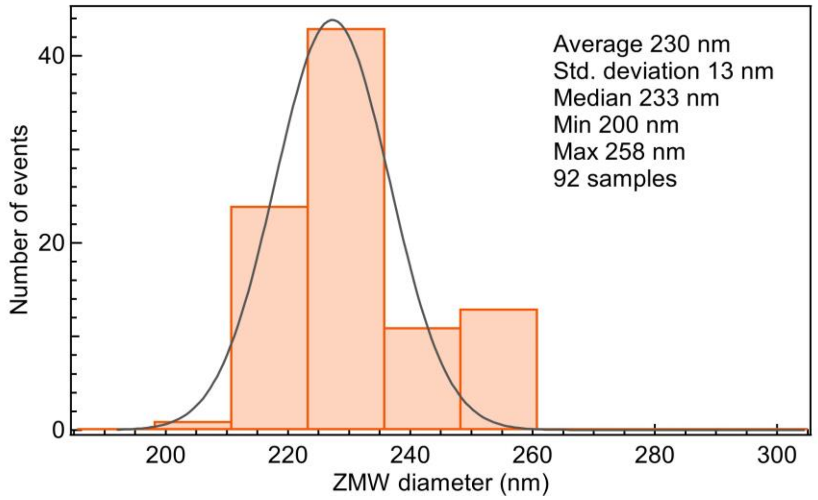

Figure S3. Histogram of the ZMW diameter determined from SEM images of 92 individual structures analyzed with ImageJ software.

S4. Optical image of the ZMW array

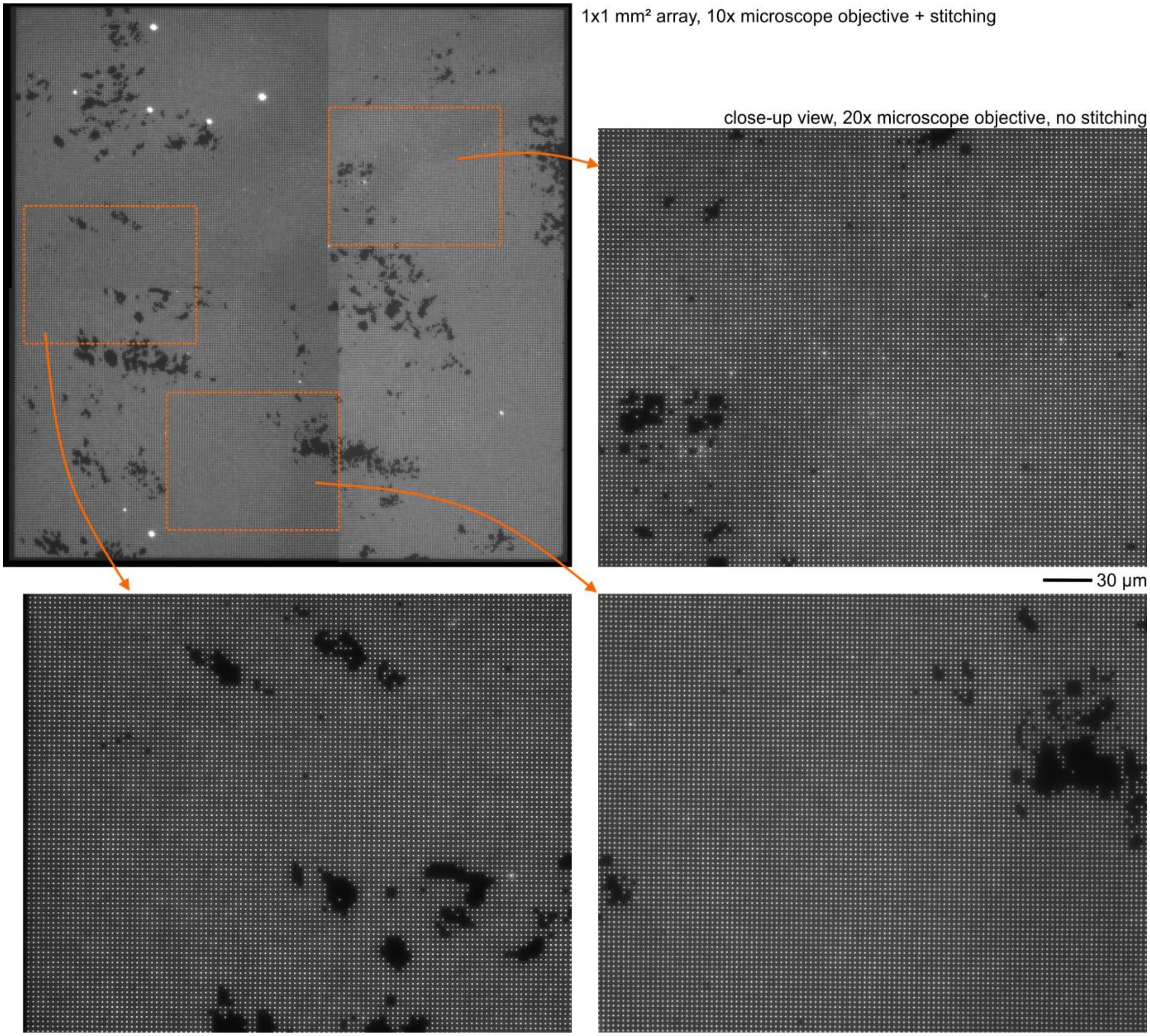

Figure S4. Optical image of the total 1 x 1 mm² array fabricated using our process. 4 individual images taken with a 10x microscope objective were stitched together to produce the final image of the total array, hence the slight variations in gray levels between quarter zones on the reconstructed image. Black areas are defects due to non-imprinted pillars during the NIL process. These can serve as fiducial marks to navigate across the sample and re-observe the same area. The 3 close-up view images are recorded with a 20x magnification objective without further processing. Each image covers a 330 x 265 µm² field of view. Low magnification objectives were used here to show field of views as large as possible. Typical fluorescence microscopy experiments would use a high numerical aperture with a larger magnification and smaller field of view.

S5. Nanoimprint of pillars with smaller diameters

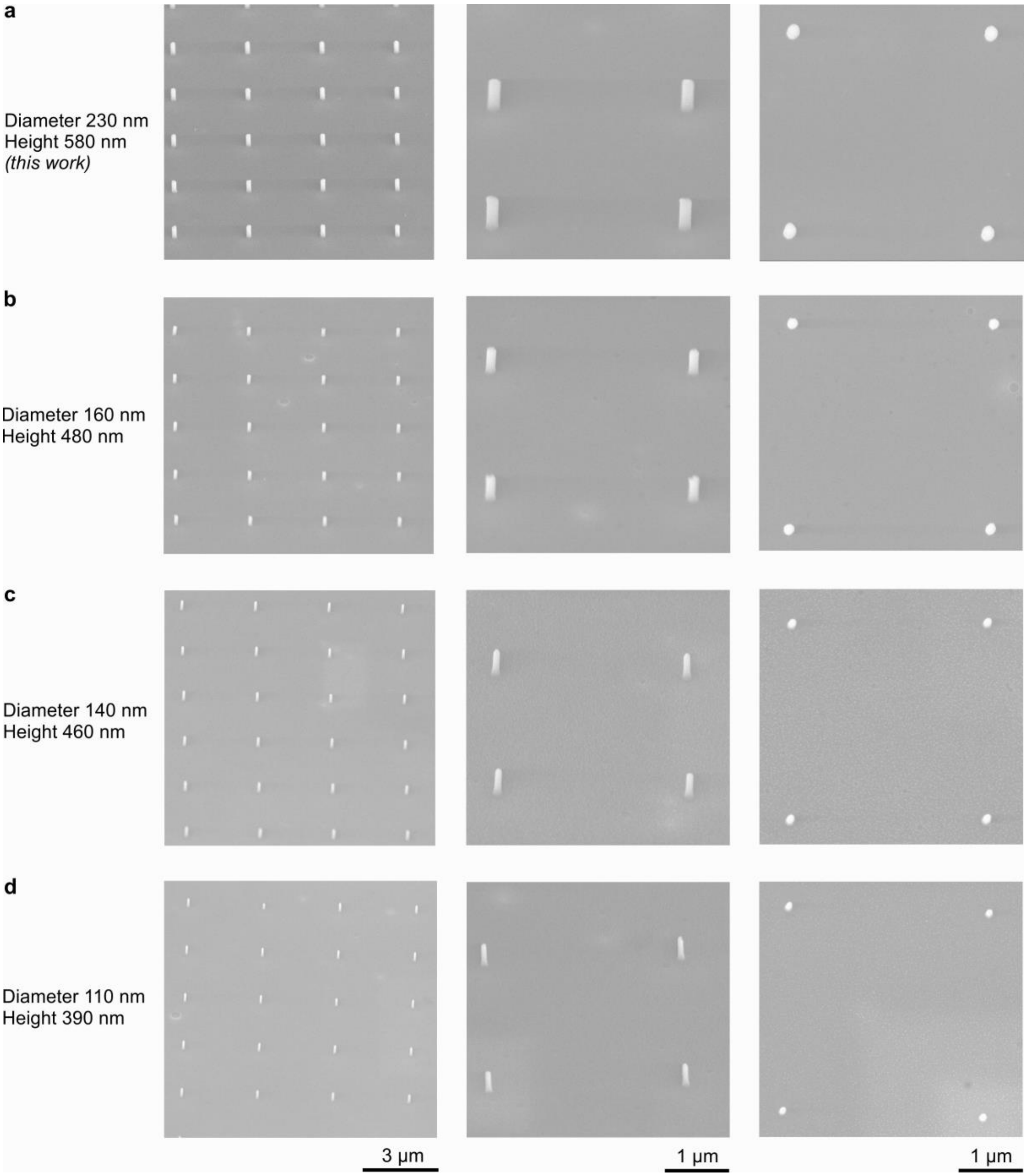

Figure S5. Demonstration that SiO_2 nanopillars of diameters down to 110 nm can be imprinted using our approach.

S6. Fluorescence time traces showing single molecule bursts

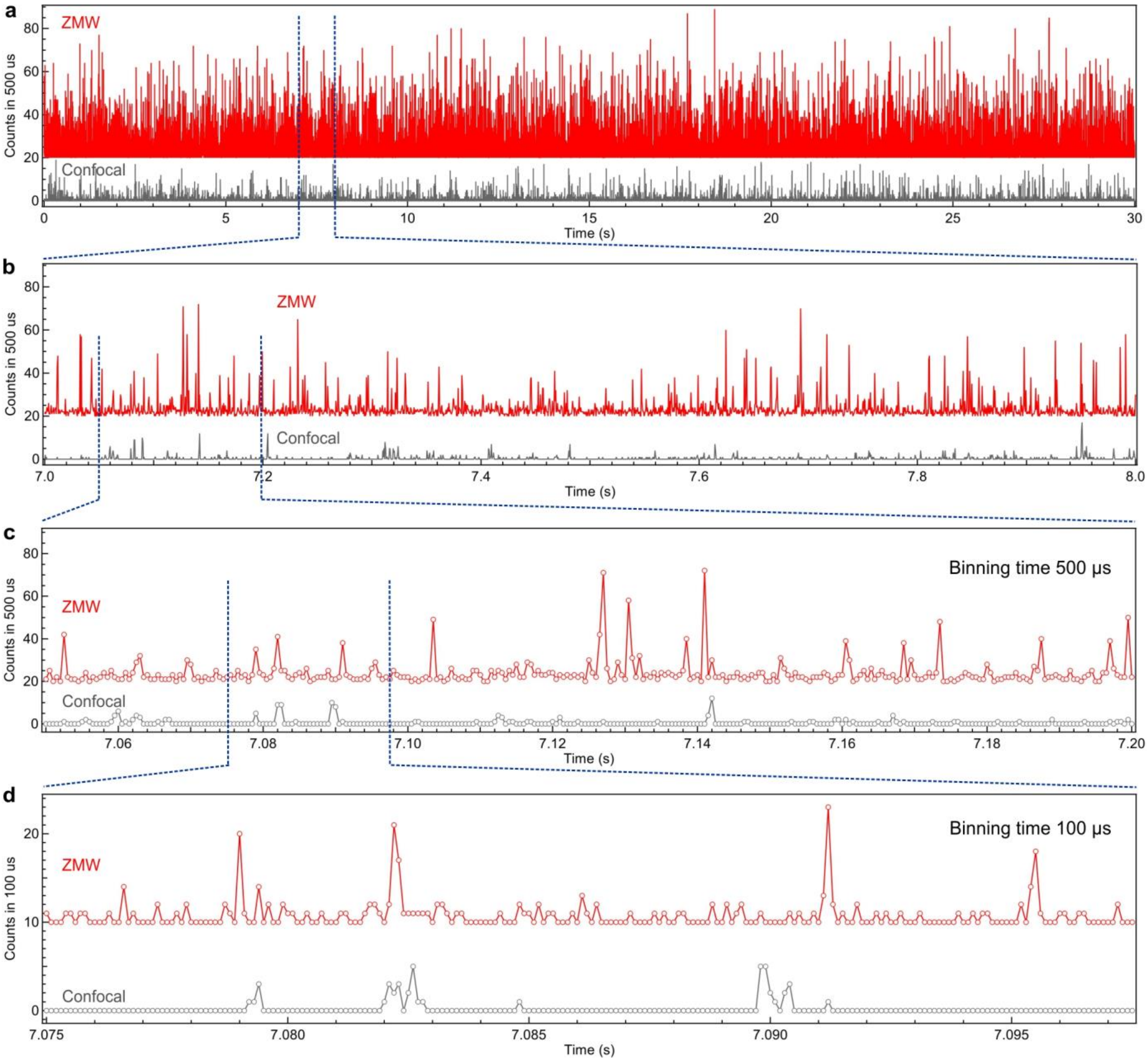

Figure S6. Extended views of the time traces shown in Fig. 2c for diffusing single Alexa Fluor 647 molecules in the ZMW and in the diffraction-limited confocal detection volume. For (a-c) the binning time is 500 µs, while 100 µs binning was used in (d) to highlight the higher signal and temporal resolution achievable with the ZMW. The time traces for the ZMW are shifted vertically by +20 counts / 500 µs in (a-c) and by +10 counts / 500 µs in (d) to enable a better viewing.

S7. Acceleration of the total integration time required to record 1000 single molecule bursts

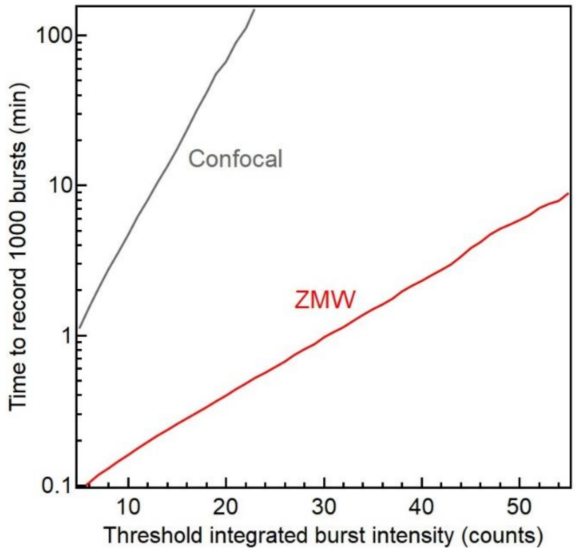

Figure S7. Total integration time needed to record 1000 fluorescence bursts in the conditions of Fig. 2c as a function of the intensity threshold used for the burst detection. The data is computed from the events frequency obtained in Fig. 2f. Thanks to a higher signal, shorter diffusion time and improved detection efficiency, the use of ZMWs enables a reduction of the total integration time by over 2 orders of magnitude as compared to the diffraction-limited confocal configuration.

S8. Influence of HF etching time

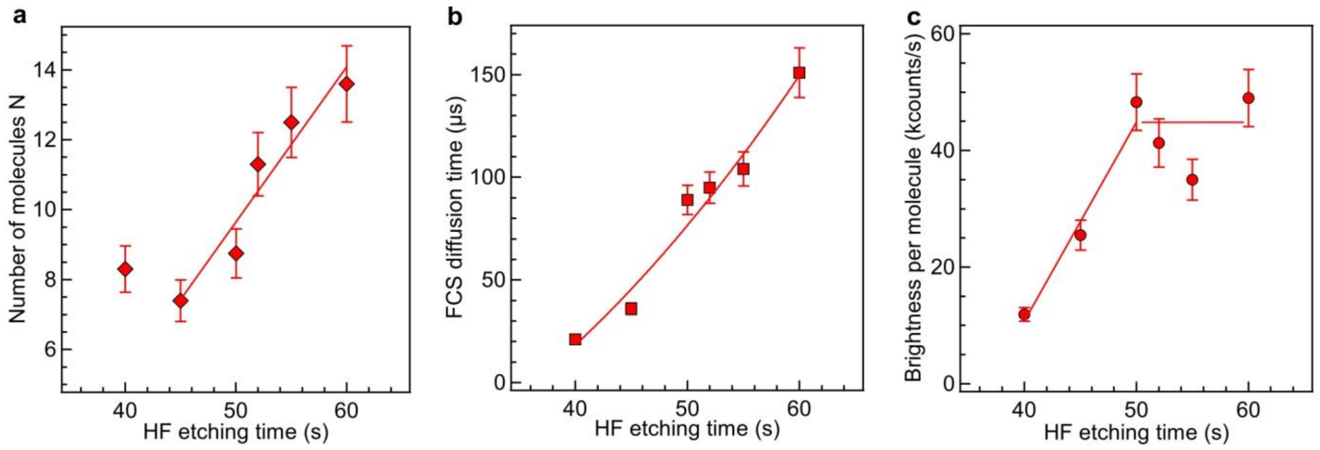

Figure S8. Dependence of the FLCS parameters on the HF etching time: (a) number of molecules N detected in the ZMW volume, (b) diffusion time τ_d and (c) brightness per molecule CRM. All the experiments were performed in conditions similar to those of Fig. 2 and 3, with Alexa Fluor 647 molecules at 1.4 µM concentration and 10 µW laser power. The data points are averaged values over at least 6 different ZMW apertures, the error bars represent one standard deviation.

S9. Statistical dispersion of the data

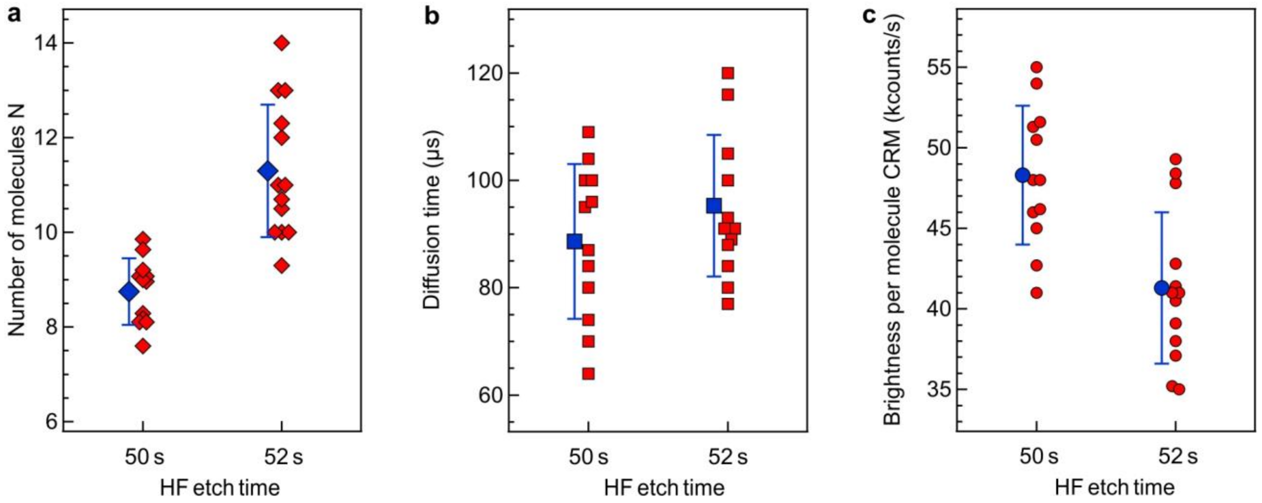

Figure S9. Scatter plots of the FLCS parameters recorded on 12 different ZMWs on two different samples with 50 or 52 s HF etching time. (a) Number of molecules N detected in the ZMW volume, (b) diffusion time τ_d and (c) brightness per molecule CRM. Each red data point is recorded on a different ZMW aperture. Large blue markers with error bars represent the average value and +/- one standard deviation. Some data are slightly horizontally shifted to enable a better viewing. The Alexa Fluor concentration is 1.4 µM and the excitation power is 10 µW. Typically, the experimental uncertainties amount to 10% for N and CRM, and 15% for τ_d. These errors include the ZMW positioning accuracy respective to the laser focus and the uncertainty in the FLCS data interpolation. No post-selection nor filtering was applied to the data.

S10. Re-usability of the sample

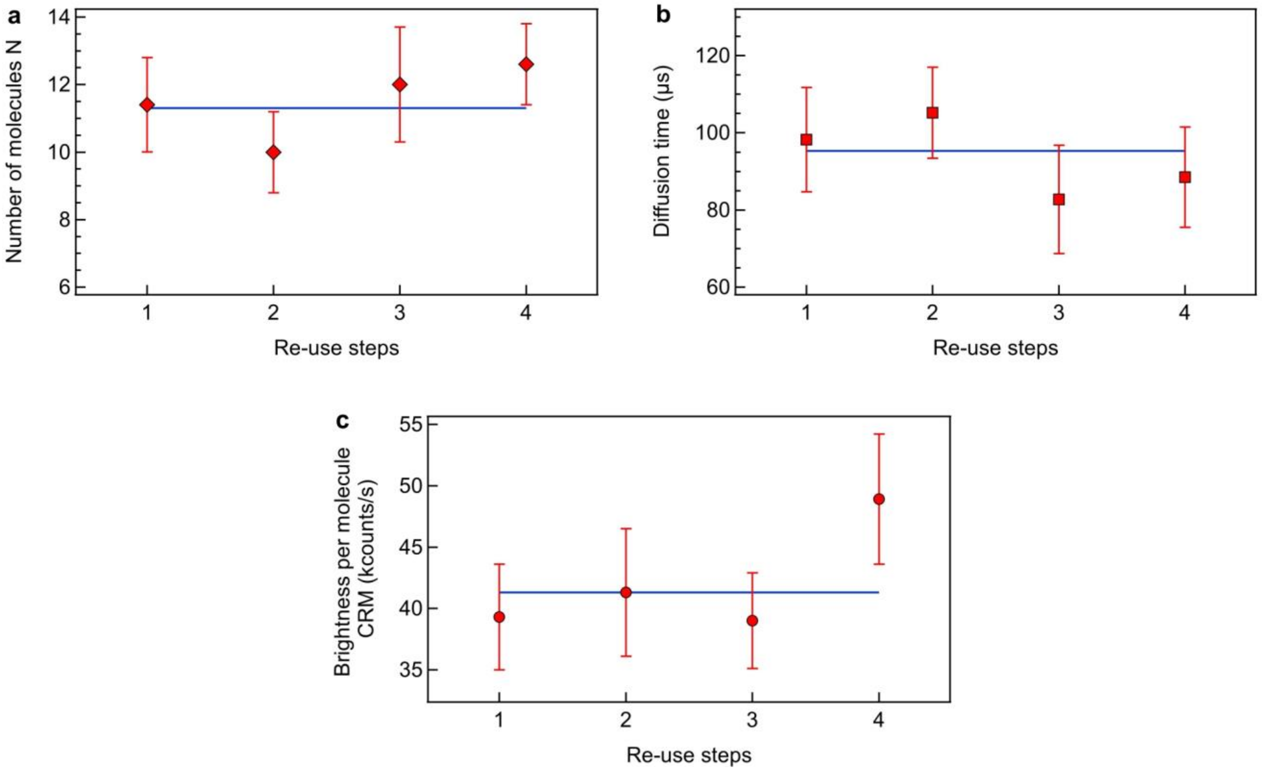

Figure S10. Demonstration of the re-usability of the ZMW sample (52 s HF etching time, corresponding to data in Fig. S6) after several washing and cleaning steps, leading to statistically similar results. (a) number of molecules N detected in the ZMW volume, (b) diffusion time τ_d and (c) brightness per molecule CRM. As for Fig. S5 and S6, the Alexa Fluor concentration is 1.4 µM and the excitation power is 10 µW. The data points are averaged values over at least 6 different ZMW apertures, the error bars represent one standard deviation. A typical washing and clean step corresponds to (i) rinsing the sample with pure water to remove the fluorescent solution and the salts in the buffer, (ii) rinsing with 70% ethanol-water solution, (iii) drying with nitrogen and (iv) 6 minutes exposure to UV-ozone cleaning system (Novascan PSD200).

S11. Raw FCS data for dilution series on different dyes

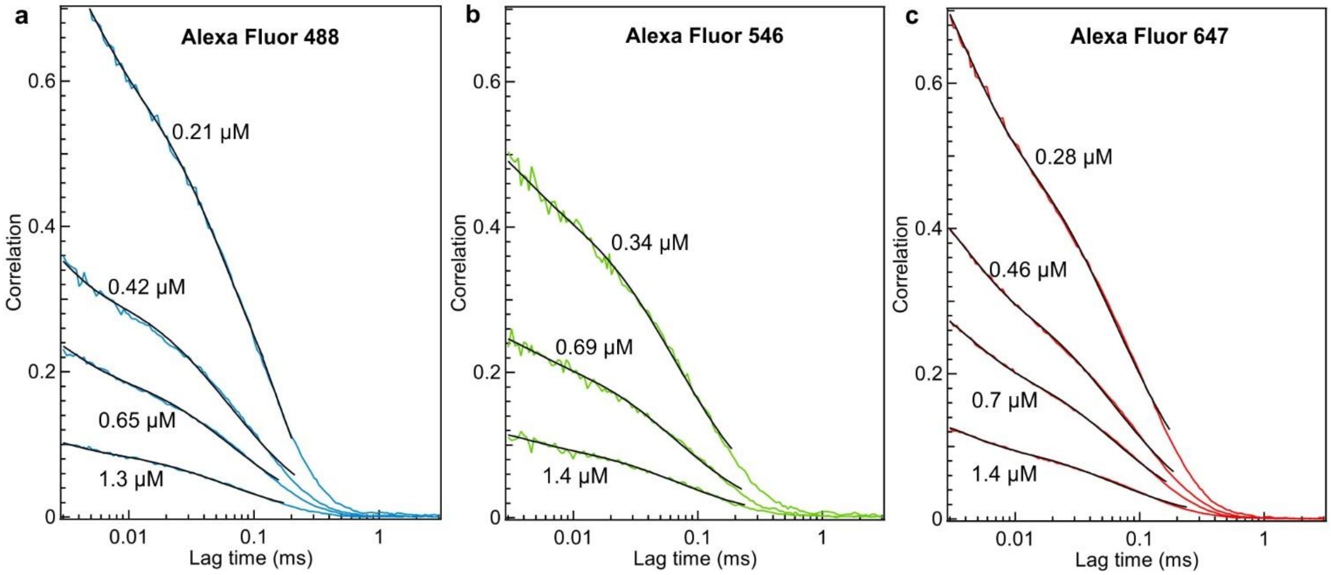

Figure S11. Raw FCS data used to derive Fig. 3j, performed on the same ZMW aperture at 10 µW laser power (linear excitation regime) for Alexa Fluor 488 (a), 546 (b) and 647 (c). Black lines are numerical fits using the model equation (1) with s=1 and limiting the fitting range from 2 µs to 200 µs. The concentration of the stock solution of each dye was determined using Tecan Spark10M absorbance spectrophotometer. The integration time was 50 s for AF488, 60 s for AF546 and 30 s for AF647.
